# Household cost of out-patient treatment of Buruli ulcer in Ghana: a case study of Obom in Ga South Municipality

**DOI:** 10.1186/1472-6963-13-507

**Published:** 2013-12-05

**Authors:** Hannah Brown Amoakoh, Moses Aikins

**Affiliations:** 1 Senior Medical Officer, Department of Civilian and Military Polyclinics, 37 Military Hospital, Accra, Ghana; 2 Department of Health Policy, Planning & Management, School of Public Health, College of Health Sciences, University of Ghana, P. O. Box LG13, Legon, Accra, Ghana

**Keywords:** Buruli ulcer, Economic treatment cost, Cost burden, Social isolation, Ghana

## Abstract

**Background:**

The economic burden of diseases has become increasingly relevant to policy makers as healthcare expenditure keep rising in the face of limited and competing resources. Buruli ulcer (BU), a neglected but treatable tropical disease caused by *Mycobacterium ulcerans*, the only known environmental mycobacterium is capable of causing long term disability when left untreated. However, most BU studies have tended to focused on its bacteriology, epidemiology, entomology and other social determinants to the neglect of its economic evaluation. This paper reports estimated the household economic costs of BU and describe the intangible cost suffered by BU patients in an endemic area.

**Methods:**

Retrospective one year cost data was used. A total of 63 confirmed BU cases were randomly sampled for the study. Economic cost and cost burden of BU were estimated. Sensitivity analysis was conducted to test the robustness of the cost estimates. Intangible cost measured stigmatization, pain, functional limitation and social isolation of children.

**Results:**

The annual total household economic cost was US$35,915.98, of which about 65% was cost incurred by children with a mean cost of US$521.04. The mean annual household cost was US$570.09. The direct cost was 96% of the total cost. Non-medical cost accounts for about 97% of the direct cost with a mean cost of US$529.27. The mean medical cost was US$18.94. The main cost drivers of the household costs were transportation (78%) and food (12%). Caregivers and adult patients lost a total of 535 productive days seeking care, which gives an indirect cost valued at US$1,378.67 with a mean of US$21.88. A total of 365 school days (about 1 year) were lost by 19 BU patients (mean, 19.2 days). Functional loss and pain were low, and stigma rated moderate. Most children suffering from BU (84%) were socially isolated.

**Conclusion:**

Household cost burden of out-patient BU ulcer treatment was high. Household cost of BU is therefore essential in the design of its intervention. BU afflicted children experience social isolation.

## Background

Buruli ulcer (BU), is a neglected infectious disease of tropical and subtropical climates caused by *Mycobacterium ulcerans*, the only known environmental mycobacterium and the third commonest mycobacterium after *Mycobacterium tuberculosis* and *Leprosae.* BU is capable of causing long term disability when left untreated but though thoroughly researched, has limited studies that evaluate its economic burden [1-4]*.* The precise distribution and prevalence of BU is unknown, but it is reported annually in over 30 countries globally, the highest burden being in Sub-Saharan Africa [4,5]. BU can affect people of all ages but typically affects children less than 16 years and living in swampy, rural impoverished communities with limited access to healthcare [3,4,6,7]. Though the reservoir and mode of transmission of *Mycobacterium ulcerans* remains obscure, antecedent trauma has been implicated and living in a BU endemic area is the most important risk factor for acquiring BU [4,7-10]. *Mycobacterium ulcerans* secretes mycolactone a powerful toxin that causes massive cutaneous tissue destruction and immunosuppression [4,9]. BU presents as active disease of ulcerative or non-ulcerative forms, or as inactive disease characterized by a deep star shaped scar [4]. Recent use of rational antibiotic therapy has improved clinical outcomes of BU limiting surgery to large ulcers [7,11,12].

Ghana, the second BU most endemic country in the world reports about 1,000 cases of BU annually [4,13]. Whilst knowledge about the aetiology of BU has improved in affected Ghanaian communities, there is still late reporting of the disease from fear of limb amputations, prolonged hospital stay, transportation and treatment costs at health facilities, loss of earnings and stigmatization of BU patients [1,14]. Poor implementation of free BU treatment services has led to inconsistent service provision. However, free BU services excludes transportation, feeding or accommodation costs of patients and their caregivers - a cost that has significant implications to household economies [3,15,16]. There is however, paucity of data on the cost and the burden of BU on affected households in Ghana as well as intangible costs borne by the patients. Though the indirect cost of BU was estimated at $550 with 265 productive days lost among ulcerative cases of BU in previous Ghanaian studies, the total cost (direct and indirect) and the economic burden of BU to household is unknown [6,17]. The economic burden of diseases has become increasingly important as healthcare expenditure rapidly escalates whilst resources remain limited. This study was conducted to estimate the household economic costs of BU and describe the intangible cost suffered by BU patients in an endemic area.

## Methods

### Study area

The study was conducted in the Obom sub-district of the Ga South Municipality, Greater Accra Region, Ghana in May 2012. The first 5 districts nationwide with the highest prevalence of BU are: Amasie West (150.8/100,000), Asante Akim North (131.5/100,000), Upper Denkyira (114.7/100,000), Afigya Sekyere (107.1/100,000) and Ga (87.7/100,000) [1,2]. Thus the study district is the fifth BU most endemic area in Ghana (i.e., prevalence of 87.7/100,000) and it has the highest burden of BU related deformity and disability. Obom has 362 communities with an estimated population of 210,727 and most adults are engaged mainly in small scae agricultural activities. The rural and swampy setting of Obom increases the risk of acquiring BU. The Obom Health Centre (OHC), the main centre for BU treatment has no BU ward and therefore runs only an out-patient BU clinic. The OHC also runs Community based Health Planning and Services (CHPS) compounds that provide healthcare services in the communities. A non-governmental organization (NGO), “Stop Buruli Project” assists OHC by providing transport fare, breakfast and some logistics to the OHC and patients.

### Study design

**Figure 1:**
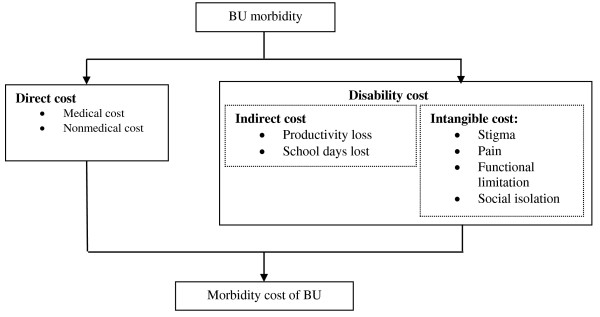
Conceptual framework of household BU morbidity costs.

The study was a cross-sectional Cost-of-Illness study (COIs) from the household perspective. Economic costs were considered as shown in Figure 1. BU morbidity costs associated with treatment service were classified broadly into direct cost and disability costs [15]. The direct costs were further categorized as *i)* Medical costs included wound dressing supplies and other treatments (i.e., medications and other laboratory tests). *ii)* Nonmedical cost included transportation costs and feeding costs for patients and caregivers and miscellaneous costs. The bottom-up approach of direct cost estimation was used [16]. The disability costs consisted of indirect costs and intangible costs. The indirect costs were productivity loss and school days lost by household members due to BU. Productivity losses were valued using the human capital approach [18-21] based on the actual local daily wage of working adults. Productivity losses of unemployed adults and children were not valued. Casual work was assumed for all working adults. Finally cost burden incurred by households were estimated. The local cost in Ghanaian currency (i.e., Ghana Cedis) was converted to their US dollar equivalents using the average exchange rate during the study period. Intangible costs were measured by describing the stigma, pain functional limitation and social isolation experienced by BU sufferers [14,15,22-24]*.*

The number of school absenteeism days due to BU was operationally measured as non-attendance of a full school day session, and calculated as the summation of the number of school days lost by BU patients and their care givers of school going age, but was not valued in monetary terms as children below the age of 18 years in Ghana by law, are not employable.

### Study population and sample size

Households with confirmed cases of BU at the OHC from 18^th^ May 2011 to 17^th^ May 2012 constituted the sample frame. An initial 105 confirmed cases was obtained from OHC database. However, 3 cases were confirmed dead and 3 others were conveniently sampled for pre-testing the questionnaire bringing the final sampling frame to 99 confirmed cases. Using BU prevalence in the area as 87.7/100,000 in 2002 [2] and a precision of 0.8% at 95% confidence interval, the sample size was calculated using the Cochran’s formula [25]:

*n = (t*^2^* p(1-p))/d^*2*^, where *n* is sample size, *t* is 1.96, *p* is 0.000877 and *d* is 0.8% - a sample size of 52.6 was obtained. The sample was further increase by a 20% non-response rate and/or recording errors, and rounded up to 63. Thus, a total of 63 confirmed BU cases and their households were randomly sampled for the study.

### Data collection

Respondents were followed into their communities and a structured questionnaire was used to collect data on the patients’ socio-demographic characteristics, household income, direct, indirect and intangible costs associated with seeking BU treatment services the last 12 months. Telephone interviews were used to clarify responses after initial data collection. The most reported intangible cost of BU are stigmatization, pain, functional limitation and social isolation [14,15,22-24]. Responses to queries on functional limitation and stigma were collected on a 5 point Likert scale [26]. Self reported felt stigma was studied using Vlassoff queries and modified for respondents who were children [24]. Data on functional limitation was collected using the Buruli Ulcer Functional Limitation Score Questionnaire (BUFLSQ). Functional limitation was defined as impairment in carrying out daily activities as a result of BU [22,23]. Daily activities listed in the BUFLSQ irrelevant to a BU patient (either because the patient is too young or old to perform the task), was recorded as not applicable. Self-reported ability to perform activities listed on the BUFLSQ was recorded without respondents being required to perform the tasks [22,23]. Self-reported perceived BU related pain was measured using a modified short MacGill pain questionnaire for children older than 11 years [27]. Social isolation was measured as the frequency a child is accompanied to the OHC by a caregiver during treatment and operationally defined as a child not being accompanied by a caregiver 50% of the time to the BU facility when seeking care as by culture, in Ghana, children are accompanied by caregivers when seeking medical care.

### Statistical and data analysis

Data was entered in Microsoft Excel 2007 and STATA Version 10.0 was used for most of the analysis. The unit of analysis was BU affected households. The total household economic cost (i.e., medical, non-medical and indirect costs), productive days lost, mean costs and confidence intervals were estimated. Tables 1 and 2 provide details of the cost and productive lost estimations and valuation. The economic cost burden was estimated as a percentage of the household cost of UB treatment divided by the total household income. Sensitivity analysis was conducted to determine the robustness of cost estimation.

**Table 1:** Household direct costs of treating BU, Ghana, 2012

**Direct costs**	**Type of cost**	**Cost (US$ per year)**
Medical	Wound dressing supplies	Summation of the cost of all wound dressing supplies used during treatment per month and normalized by sample size.
	Other treatments	Summation of all out-of-pocket payments incurred on drugs and laboratory tests by BU patients at OHC per month and normalized by sample size.
	Medical cost	Summation of total costs of wound dressing supplies and other treatments.
Non-medical	Transportation	Summation of the number of visits made by the BU patient and/or with caregiver to OHC per week multiplied by the return fare and normalized by sample size.
	Food	Summation of the cost of food bought for the patient and/or caregivers during BU treatment at OHC per week multiplied by the number of BU patients and normalized by sample size.
	Miscellaneous	Summation of costs of phone calls made, cost of extra soap and disinfectants used for washing of soiled clothing during BU treatment by all patients and normalized by sample size.
	Nonmedical cost	Summation of the total costs of transportation, food and miscellaneous.
	Total direct cost	Summation of total medical and non-medical costs.

**Table 2:** Household indirect costs of treating BU, Ghana, 2012

**Indirect costs**	**Time**	**Estimation approach**
Travel time	Patient	Summation of the product of the number of visits by BU patients to OHC per week by doubled travel time (i.e., to and fro OHC) and normalized by sample size.
	Caregiver	Summation of the product of the number of visits by caregivers to OHC per week by doubled travel time (i.e., to and fro OHC) and normalized by sample size.
	Total travel time	Summation of patient and caregiver travel times.
Wound dressing time	Patient	Summation of the product of time spent on wound dressing (i.e., waiting and dressing) by the number of wound dressing times per week by the number of weeks of wound dressing and normalized by sample size.
	Caregiver	Summation of the product of the caregiver’s waiting time spent on wound dressing by the number of wound dressing times accompanied by caregiver per week by the number of weeks of wound dressing accompanied by caregiver and normalized by sample size.
	Total wound dressing time	Summation of patient and caregiver travel times.
**Valuation**	**Type of cost**	**Cost (US$ per year)**
	Productivity lost by patient	Summation of the product of the total number of workdays lost by adult BU patients in seeking health care (i.e., travel and wound dressing times) by the average local daily wage rated.
	Productivity lost by caregiver	Summation of the product of the total number of workdays lost by caregivers in seeking health care (i.e., travel and wound dressing times) by the average local daily wage rated.
	Total indirect cost (i.e., productivity lost)	This is the summation of valued productive days lost by patient and caregiver.

The main intangible costs measured were stigmatization, pain, functional limitation and social isolation. An overall mean for stigma, pain and functional limitation was computed as the mean of the means of each Likert scale item for children and adults separately. The Mann–Whitney test was used to compare Likert scale responses among male and female. A p-value < 0.05 was considered significant for Mann–Whitney tests. Social isolation was calculated as the proportion of children who were not accompanied to the OHC during treatment.

The main assumptions made were: *i)* Costs estimated were incurred during the period, and were a consequence of BU; *ii)* Household income prior to the onset of BU would have remained constant over the 12 months study period had BU not afflicted a household member; and *iii)* Children did not work and so did not incur productivity loss.

### Ethical review

Ethical clearance for the study was obtained from the Ethical Review Committee, Research and Development Division of the Ghana Health Service, Ghana. Study approval was also obtained from the Ga South Municipal Health Administration and the Obom Health Centre management. Identified respondent/caregiver was informed about the study. Respondent/caregiver was at liberty to withdraw from the study at will. Respondent/caregiver was assured of all information collected. Consented participant/caregiver was interviewed alone and in privacy. No compensations were paid to them. Written informed consent was obtained from all respondents.

## Results

### Socio-demographic characteristics of the study population

Table 3 shows that 52% of the BU cases were male and about 62% of BU cases were less than 15 years old with a median age of 13 years. Eighty-four percent of them reported being Christians. About 74% had primary school education of which 43% were male. Majority of respondents (75%) were students/apprentices and 77% lived in household size of 5 or more persons. Majority of respondents (87%) had one lesion on their body with no difference between the sexes. All households studied had one household member suffering from BU except one household that had 2 BU sufferers. The mean and median duration of treatment was 3 months each.

**Table 3:** Socio-demographic characteristics of BU cases

**Background characteristic**	**Male (%)**	**Female (%)**	**Total (%)**
**Age (years)**			
<15	23 (37)	16 (25)	39 (62)
15 and over	10 (16)	14 (22)	24 (38)
**Religion**			
Christian	27 (43)	26 (41)	53 (84)
Other^†^	6 (2)	4 (7)	10 (17)
**Educational background**			
No education	1 (2)	5 (8)	6 (10)
Primary	27 (43)	20 (32)	47 (74)
Middle/JHS/JSS*	5 (8)	5 (8)	10 (16)
**Occupation**			
Student/apprentice	27 (43)	20 (32)	47 (75)
Other	6 (10)	10 (16)	16 (15)
**Household Size**			
<5	4 (6)	10 (16)	14 (22)
5 and over	29 (46)	20 (31)	49 (77)
**Number of Buruli Ulcer lesions**			
1	28 (44)	27 (43)	55 (87)
2 and over	5 (8)	3 (5)	8 (13)
Total	33 (52)	30 (48)	63 (100)

*JHS and JSS means Junior High School and Junior Secondary School respectively.

^†^Other includes Muslim, Traditional and Other minor religions.

### Total and mean household economic costs

The annual total household economic cost was US$35,915.98, of which about 65% was cost incurred by children with a mean cost of US$521.04. The mean annual household cost was US$570.09. The direct cost was 96% of the total cost. Non-medical cost accounts for about 97% of the direct cost with a mean cost of US$529.27. The mean medical cost was US$18.94. The main cost drivers of the household costs were transportation (78%) and food (12%), with a mean cost of US$444.71 and US$69.76 respectively. For both children (p = 0.11) and adults (p = 0.89) there was no significant difference in household cost among the sexes. Table 4 provides the total and mean household economic costs.

**Table 4:** Estimated total household economic cost of BU, Ghana, 2012

**Direct costs**	**Economic cost (US$)**			**Cost profile (%)**
	**Children (n = 45)**	**Adults (n = 18)**	**Total (n = 63)**	
Medical cost:				
Wound dressing supplies (i.e., bandages, gauze, disinfectants, vaseline)	504.32 (11.21)^†^	606.79 (33.71)	1,111.11 (17.64)	3.1
Other treatments (i.e., medication & laboratory tests ):	53.70 (1.19)	28.52 (1.58)	82.22 (1.31)	0.2
Sub-total:	558.02 (12.40)	635.31 (35.30)	1,193.33 (18.94)	3.3
Non-medical cost:				
Transportation	18,866.67 (419.26)	9,150.33 (508.35)	28,017.00 (444.71)	78.1
Food (i.e., patient & caregiver)	3,094.63 (68.77)	1,300.00 (72.22)	4,394.63 (69.76)	12.2
Miscellaneous (i.e., soap, clothes, disinfectants, phone calls etc.)	594.69 (13.22)	337.66 (18.76)	932.35 (14.80)	2.6
Sub-total:	22,555.99 (501.24)	10,787.99 (599.33)	33,343.98 (529.27)	92.8
Total direct cost	23,114.01 (513.64)	11,423.30 (634.63)	34,537.31 (548.21)	96.2
Total valued indirect cost (i.e., productivity lost)	332.98* (7.40)	1,045.69 (58.09)	1,378.67 (21.88)	3.8
Total cost (direct + indirect costs)	23,446.99 (521.04)	12,468.99 (692.72)	35,915.98 (570.09)	100

*Caregivers associated cost not children.

^†^Figures in parenthesis are means.

Caregivers and adult patients lost a total of 535 productive days seeking care, which gives an indirect cost valued at US$1,378.67 with a mean of US$21.88. A total of 365 school days (about 1 year) were lost by 19 BU patients (mean, 19.2 days). Though most children were able to go to school after receiving treatment, about 33% [15] of them reported late to school due to time spent seeking care at the facility. No BU caregiver was schooling. The household economic cost of BU treatment constituted about 45% of the household annual income.

### Intangible costs

**Figure 2:**
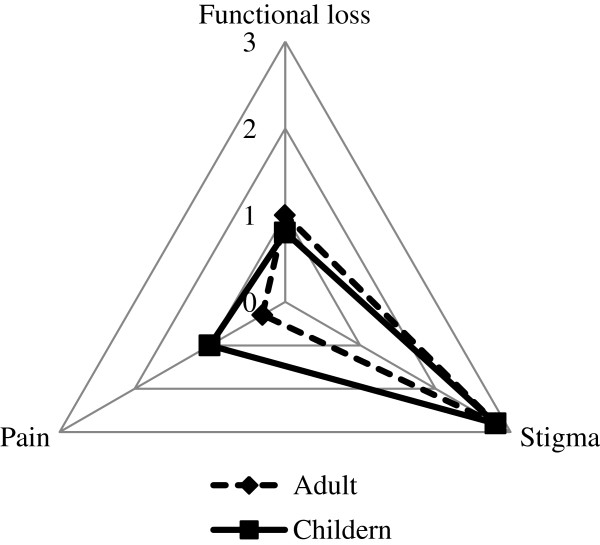
Mean responses of BU adults and children.

About 92% of BU patients felt some form of pain during the course of BU. Perceived pain and functional loss was low among respondents. Children felt more pain (mean, 1.0 and 0.3 for children and adults respectively) but had less functional loss compared to adults (mean, 0.8, 1.0 for children and adults respectively) as shown in Figure 2.

The mean response of both adult and children BU cases to stigma queries was 2.8. BU afflicted men were more likely to indicate that others think less of them (p = 0.045). There was no difference between male and female responses to other queries on stigma, perception of pain or functional loss. About 84% of children were socially isolated, with 36% of these children never being accompanied for treatment. Those who were accompanied throughout their treatment were 11%. The most cited reasons for this phenomenon were that the children could go by themselves and lack of money for transportation.

## Discussion

The objective of this COIs study was to estimate household cost of BU in an endemic area. The distribution of age, sex and location of BU lesions among respondents is consistent with other BU studies [4,6,10,13]. The mean cost of BU treatment of US$570.09 represents about 17% of Ghana’s 2012 Gross Domestic Product (GDP) per capita of $3,300^a^ (PPP). The high direct cost reported is in keeping with previous studies on healthcare costs [16,18]. High contribution of non-medical direct costs mainly in the form of transportation was also found in Hong Kong and is explained by, the use of out-patient study participants who required daily transportation to the health facility for treatment [20]. We report lower food cost compared with that of other chronic illness like tuberculosis and Human Immunodeficiency Virus as BU patients do not typically require a special diet [16]. A lower direct cost of BU in a previous Ghanaian study is due to categorization of food and miscellaneous costs as indirect cost as compared to this study [6]. Low medical cost in our study stems from the free and short duration of treatments (median, 3 months) offered [6]. Other drivers of direct costs like co-morbidities and in-patient costs were not considered in this study [28].

High indirect cost of BU has been documented in Ghana and Cameroon among hospitalized patients whose caregivers rented accommodation, or regularly visited them [6,15]. We report lower indirect cost in this study because- our respondents were resident at home, most BU patients were students (75%) whose productivity loss was not valued, most children were either never accompanied (36%), or accompanied less than 50% of the times (48%) to the OHC for treatment- their caregivers could therefore continue with their daily activities and hence incurred lower productivity losses (mean annual indirect cost, US$7.04). Very high indirect cost (about 80% of total cost) documented among Swedish patients stems from the sole use of study participants in the productive age group in that study [21].

This study confirms that BU treatment poses significant burden on households forming a 45% economic burden. BU affected households are therefore likely to become impoverished. Despite the high cost burden of BU, affected households may be coping in the Ga South Municipality due to financial assistance with transportation and food costs from the NGO operating at the OHC. Communities that receive no such assistance are likely to develop coping mechanisms like sale of assets, borrowing of money and abandoning health facility based treatment documented in other studies [15,17]. Sensitivity analysis showed the estimated treatment costs were robust because varying assumptions and uncertainty causes a minimal 2.1% change in total BU costs.

School days lost by children in this study (mean of 19 days) is lower than reported in Cameroon [15]. The observed difference may be a consequence of choice of study participants - hospitalized patients who could obviously not be in school in the Cameroonian study, versus non-hospitalized patients who came for treatment and went to school afterwards in our study. Hospitalizations of BU patients suggest late presentation of BU requiring lengthier treatment and as such loss of more school days as compared with out-patient participants in this study. This implies that child BU patients in this study reported to the facility with early stages of BU. Such early reporting can be explained by intensification of health education and surveillance [29].

Buruli ulcer associated stigma is moderate among study participants (mean, 2.8 for both adults and children). Median scores for stigma among BU sufferers were computed using a four point Likert-like scale in an earlier study [24]. Acceptability of BU in this study community may be because stigma is reportedly low in highly endemic areas and where the cause of BU is not ascribed to magico-religious beliefs [14,24]. A preference to keep others from knowing they have BU despite acceptability and sympathy for BU patients, and lack of significant difference in male and female response to stigmatizing behaviour we report, is consistent with an earlier finding [14]. High levels of discrimination of BU children among their peers reported earlier in Ghana was observed to be absent in this study - an attitude change attributable to increasing levels of education about BU in schools in endemic areas [14,24].

Buruli ulcer is minimally painful when it presents as an oedematous lesion and this explains a general reporting of mild degree of pain in this study [4]. Functional limitation of BU patients in this study is described using a 5 point Likert scale which contained 19 items of the BUFLSQ. Previously reported BU related functional loss was done using a 3 point Likert-like scale and computing a functional loss that was a percentage of activities that a BU sufferer could no longer perform (as a result of BU) out of the 19 items of the BUFLSQ applicable to the BU sufferer [22,23]. The low functional limitation documented in this study is due to the selection of study participants who generally had less extensive lesions and therefore were seen on out-patient basis as compared to hospitalized patients who were likely to have more extensive lesions requiring surgery in the previous studies [22].

Most children (84%) are socially isolated in this study. Social isolation as a coping mechanism for high BU cost of treatment was reported in Cameroon [15]. The higher rate of social isolation among children in this study as compared with the Cameroonian study (63% of households isolated their BU household member) can be explained by not just high BU treatment cost but also by trust of caregivers in motorbike riders who are mostly locals of the communities contracted by the OHC and the operating NGO to transport all confirmed cases to the OHC and back. The social isolation of the children in this study may however, affect compliance with treatment as caregivers are not present to ensure compliance. This may in effect prolong treatment and further increase treatment costs.

### Limitations of the study

The main limitation of this study is about the sample which is facility-based rather than population based and this can lead to selection bias. This data was however used because they were confirmed cases well documented and readily available. However, there is the likelihood of more unreported cases in the communities. Although intangible costs of BU like functional loss, social isolation, stigma and pain were measured, they were not valued in monetary terms as such an evaluation was beyond its scope.

## Conclusion

This study has shown that despite free BU health facility based treatment; households incur substantial costs seeking BU care. For non-hospitalized BU patients, transportation cost can be enormous. Household cost of BU is therefore essential in the design of BU interventions. The total household cost of BU estimated in this study can therefore serve as a baseline cost for strategic planning and budgeting for future BU programmes, informing policy makers in the setting up of a financial framework on which future cost planning for BU will be based. An economic evaluation of BU should be undertaken with larger samples in other BU endemic areas to provide a trend and variability in cost of this debilitating disease to further inform policy on BU management.

## Endnote

^a^http://www.indexmundi.com/ghana/economy_profile.html.

## Abbreviations

BU: Buruli ulcer; BUFLSQ: Buruli Ulcer Functional Limitation Score Questionnaire; CHPS: Community based Health Planning and Services; COIs: Cost-of-Illness study; OHC: Obom Health Centre.

## Competing interests

The authors declare that they have no competing interests.

## Authors’ contributions

HBA participated in the design, undertook the data collection and participated in cost analysis. MA participated in the design and participated in cost analysis. All the authors were involved in drafting, revising the manuscript, reading and approving the final manuscript.

## Pre-publication history

The pre-publication history for this paper can be accessed here:

http://www.biomedcentral.com/1472-6963/13/507/prepub
